# Metagenomic evaluation of food hygiene practices in the National School Nutrition Programme in KwaZulu Natal, South Africa

**DOI:** 10.4102/hsag.v30i0.2814

**Published:** 2025-06-11

**Authors:** Sithembile S. Madlala, Nokuthula Mchunu, Monica Dalasile, Rian Pierneef, Poovendhree Reddy

**Affiliations:** 1 Department of Community Health Studies, Faculty of Health Sciences, Durban University of Technology, Durban, South Africa; 2 National Research Foundation, Pretoria, South Africa; 3 School of Life Science, University of KwaZulu-Natal, Durban, South Africa; 4 Department of Biochemistry, Genetics and Microbiology, University of Pretoria, Pretoria, South Africa; 5 Centre for Bioinformatics and Computational Biology, University of Pretoria, Pretoria, South Africa

**Keywords:** amplified metagenomics, food contact surfaces, food contamination, food handlers, food hygiene, National School Nutrition Programme

## Abstract

**Background:**

The National School Nutrition Programme (NSNP) provides meals to schools in low-income areas in South Africa, implemented by the Department of Basic Education (DBE) with food safety monitored by Municipal Health Services.

**Aim:**

To assess compliance of school kitchens with general hygiene requirements (R638 of 2018) and detect food pathogens on food contact surfaces using amplified metagenomics.

**Setting:**

The study was conducted in quintile 1 and 2 primary schools in Vryheid, KwaZulu-Natal.

**Methods:**

A quantitative cross-sectional study assessed the safety compliance of food preparation and storage areas in 33 primary schools against national legislation standards. Fifteen samples of food contact surfaces were collected from four schools and analysed using Illumina sequencing to identify prevalent bacterial genera.

**Results:**

None of the schools possessed a Certificate of Acceptability. Significant structural issues include poor pest control, inadequate sanitary facilities, a lack of food safety training and inadequate waste management. Taxonomic analysis revealed several dominant bacterial genera, including *Pseudomonas, Stenotrophomonas, Acinetobacter* and *Pantoea*, indicating potential routes for food contamination and subsequent risks for foodborne illnesses.

**Conclusion:**

The study highlighted critical inadequacies in food preparation and storage areas requiring urgent intervention to ensure safe meal preparation. It emphasised the need for improved food safety monitoring and compliance in schools in low-income areas. Next-generation sequencing (NGS) techniques identified a broad spectrum of pathogens, offering a robust method for assessing environmental hygiene.

**Contribution:**

This study provides insights into food safety risks in the NSNP, informing policies and interventions to improve food safety and reduce foodborne illnesses in schools.

## Introduction

Modern food processing facilities are designed to reduce the risk and likelihood of producing unsafe food. However, they are still vulnerable to colonisation by bacteria from various sources such as raw materials, air or atmosphere, personnel, pests and a variety of other sources (Doyle et al. 2015). Food production and processing environments are routinely tested for the presence of pathogens, with specific focus on particular species such as *Escherichia coli* (Doyle, Otoole & Cotter 2017), *Listeria monocytogenes, Pseudomonas aeruginosa* and *Salmonella typhi.* When applying such narrow or specific approaches, other microorganisms escape detection and restricts monitoring and control of the entire microbial ecology of the food handling environment (Mendes et al. 2011). Previously, whole genome sequencing (WGS) of cultured isolates tracked specific strains of bacteria involved in outbreaks back to food processing facilities (Staciewicz, Dan Bakker & Wiedmann 2015). This highlighted the potential for next-generation sequencing (NGS) technologies for tracking the prevalence of microbial communities in the food chain. This approach has been adopted by researchers examining clinical samples from outbreaks of foodborne illness, but it is not limited to the identification of pathogenic bacterial agents in clinical samples and can be adapted in a similar manner to investigate other food chains (Baylis 2006; Doyle 2017; Doyle et al. 2015; Huang et al. 2016).

Previous studies on school nutrition programmes from different countries such as the United Kingdom and Ghana have established that foodborne disease (FBD) outbreaks in schools pose a food safety hazard (Huang et al. 2016; Kunadu et al. 2016). In South Africa (SA), schools with food programmes are situated in or near rural communities and informal settlements and usually lack basic services such as a constant electricity supply and potable water (Sibanyoni & Tabit 2019). Foodborne disease outbreaks in schools have been reported in various provinces, thus highlighting the need to monitor food safety in the National School Nutrition Programme (NSNP) (Dlova 2018). Learners in Sekhukhune, Limpopo province suffered from nausea and abdominal pain. A subsequent investigation attributed the outbreak to the supplier of NSNP food, who allegedly contravened food safety standards (Devereux et al. 2018). In addition to disrupting learning in schools, opportunistic bacterial infections can increase mortality and morbidity in children, especially those with compromised immune systems (Abushelaibi et al. 2016; Mellou et al. 2013). Implementation of food safety measures in school feeding programmes is thus crucial (Nyenje & Ndip 2013). The presence of foodborne pathogens such as *Salmonella enterica* and *E. coli* was detected on NSNP meals in SA, while faecal coliforms were detected in water in a combined school in the Eastern Cape. An epidemiological study on school FBD outbreaks reported by the National Foodborne Disease Outbreak Surveillance System in Liaoning province (China) found that primary and secondary schools reported a significant number of incidents, with 12 cases and 55 597 hospitalisations from 2011 to 2021 (Fang et al. 2022). In addition to improper processing and cross-contamination, *Staphylococcus aureus* toxin, *Bacillus cereus* toxin and *Norovirus* were the main causes (Fang et al. 2022).

To prevent FBDs in schools, it is crucial that appropriate food hygiene measures are followed, given the rise in the number of public schools serving NSNP meals to learners in South Africa (Asiegbu et al. 2016). The provision of healthy meals depends on schools having adequate infrastructure and equipment for storing and preparing meals per provisions for general hygiene requirements for food premises gazetted under South African Regulation 638 of the *Foodstuffs, Cosmetics and Disinfectants Act* (1972). A food safety risk arises from the fact that many schools do not have a designated kitchen where meals are prepared, and this lack of infrastructure may lead to the spread of foodborne illnesses (Kibret & Abera 2012).

Hygienic practices are of paramount importance in food preparation, and food handlers have an important role in preventing food contamination that may develop into FBD outbreaks (Putri & Susanna 2018). According to the Codex Alimentarius Commission (2003), poor hand hygiene is a significant risk factor in the occurrence of food contamination, and inappropriate food handling is a primary source of foodborne illnesses. The processing environment is also crucial for the risk assessment of food safety. Environmental bacteria are indicators of the processing facility’s environmental hygiene, even though they are typically not thought to pose a threat to food safety (Ferreira et al. 2014; Møretrø & Langsrud 2017). When food contact surfaces are ineffectively cleaned and disinfected, microbial cross-contamination can arise in the food preparation areas of schools (Nhlapo, Lues & Groenewald 2014). To eliminate plant debris, soil, and microbiological pollutants that collect on surfaces during food handling, cleaning and disinfection are often carried out at the end of the shift and is a component of most food processing facilities’ overall food safety procedures (Møretrø & Langsrud 2017).

Ribosomal deoxyribonucleic acid (DNA) sequencing using NGS provides a potential means through which the microbiome of the sampled environments can be tested to identify unknown, overlooked etiological and spoilage agents without having to culture the microorganisms (Huang et al. 2016). This study focused on assessing the compliance of food preparation and storage areas in primary schools offering NSNP meals to South African Regulation 638 of the *Foodstuffs, Cosmetics and Disinfectants Act* (*No. 54 of 1972*), and on evaluating the presence of bacteria on food handlers’ hands and contact surfaces. This approach offers a powerful tool in the elucidation of the microbiome present in these environments and general food hygiene in South African school feeding programmes. This study hypothesises that the use of NGS to analyse the microbiome of food contact surfaces in primary schools participating in the NSNP in South Africa will reveal significant non-compliance with South African Regulation 638 of the *Foodstuffs, Cosmetics and Disinfectants Act* (*No. 54 of 1972*) and the presence of pathogenic bacteria that are not typically detected by standard testing methods.

## Research methods and design

### Research design

A quantitative, cross-sectional design was used for this study, and random sampling was used to eliminate bias and ensure all members of the population had an equal and independent chance of being selected. A total of 33 primary schools were randomly selected from a list of 109 public schools that provide NSNP meals in Vryheid. The selected schools in quintiles one and two were approached, informed of the study and asked to participate. All schools that responded positively were accepted until the minimum sample size of 33 was reached. All the food handlers (140) of the participating schools were observed for protective clothing and hygiene practices. Samples were collected from the food contact surfaces and food handlers of the two highest and two lowest scoring schools as per the checklist below.

### Checklist data collection

A standardised checklist comprising 39 questions with ‘Yes’ or ‘No’ as the only possible answers was used to assess the condition of the food preparation and storage areas. The checklist was completed by the researcher who is a registered environmental health practitioner (EHP). The checklist was divided into seven sections: demographics, certificate of acceptability, standards and requirements for food premises, facilities on food premises, storage of food, protective clothing and food handlers. The checklist was adapted from the provisions of R638 of the Act, which are used by EHPs for the inspection of food premises and the Red Meat Abattoirs Hygiene Assessment System Checklist (Agriculture and Rural Development 2018).

### Checklist data analysis

Scoring was allocated as follows: 0 = No and 1 = Yes for the checklist. The compliance was scored as follows: Section A–B (Standards for food premises): 0–6 were non-compliant; 7–13 were partially compliant and 14–20 were compliant. Section C–D (Standards for facilities): 0–2 were non-compliant; 3–4 were partially compliant; and 5–6 were compliant. Section E-F (Standards for food handlers): 0–5 were non-compliant; 6–10 were partially compliant; and 11–14 were compliant. Descriptive statistics were calculated using Microsoft Excel and SPSS version 28.0.

### 16s ribosomal deoxyribonucleic acid sequencing using illumina sequencing technology

#### Sample collection

Samples were collected aseptically from various food contact surfaces including chopping boards and utensils in four schools, named School A-D and the dominant hand of all the food handlers in the four selected schools using the swab method (SANS 18593:2004), after food service was completed and the kitchen had been cleaned. In total, 15 (triplicate) samples were collected from food handlers and 15 from food contact surfaces. The 30 swabs were labelled, stored in ice and processed within 24 h of collection.

#### Deoxyribonucleic acid extraction of 16s ribosomal

Microbial DNA was extracted from swabs content using QIAamp DNA Microbiome Kit (Qiagen, Hilden, Germany) as described in the manufacturer’s protocol. The swabs were resuspended and vortexed in a 1ml phosphate buffer. The samples were incubated in a lysis solution composed of 20 mM Tris-HCl, pH 8.0, 2 mM sodium EDTA, 1.2% Triton X-100, plus 20 mg/mL lysozyme at 37°C for 60 min. Deoxyribonucleic acid was extracted from 500 µL of the sample, as described in the manufacturer’s protocol (Mafuna 2019). An equal volume of the sample and buffer AHL was incubated for 45 min, followed by centrifugation at maximum speed. After treatment with Benzonase and Proteinase K, the mixture was briefly spun down at low speed. Two hundred microlitres of buffer ATL (containing Reagent DX) was added to the mixture and mixed very well. The mixture was transferred into a pathogen lysis tube L, and then the pathogen lysis tube L was placed into a 2010 Geno/Grinder (SPEX SamplePrep LLC, New Jersey, United States) for 15 min at 1700 rpm (Mafuna 2019). Thereafter, the pathogen lysis tube L was centrifuged at 10 000 × g for 1 min, and the supernatant was transferred into a fresh microcentrifuge tube. Forty microlitres of Proteinase K was added to the mixture and vortexed and then incubated at 56°C for 30 min at 600 rpm in a heating block. Two hundred microlitres of buffer APL2 was added and pulse vortexed for 30 s. The mixture was incubated at 7°C for 10 min, and the tube was briefly spun. Two hundred microlitre of ethanol was added to the lysate and mixed by pulse-vertexing for 15–30 s (Mafuna 2019). The DNA was eluted using QIAamp UCP Mini spin column after several wash steps. The quality of DNA was assessed by gel electrophoresis and nanodrop.

#### Deoxyribonucleic acid amplification and amplicon library preparation

The 16S ribosomal ribonucleic acid (rRNA) gene contains nine species-specific hypervariable regions enclosed by regions of more conserved sequence. The 16S ribosomal RNA region of approximately 470 bp and 500 bp covering the V3-V4 hypervariable was amplified employing a set of commonly used primers for the analysis of bacterial communities (5’-CCTACGGGNGGCWGCAG-3’ and 5’-GACTACHVGGGTATCTAATCC-3’). Polymerase chain reaction (PCR) assay contained 5 µL of DNA as the template, 12.5 µL 2x KAPA HiFi HotStart ReadyMix (KAPABIOSYSTEMS, United States) and 5 µL of 10 µM of each primer. Polymerase chain reactions were carried out on BIO-RAD T100TM Thermal Cycler (Bio-Rad Laboratories, United Kingdom) using the following protocol: (1) an initial denaturation step performed at 95°C for 5 min followed by 30 cycles of denaturation (95°C, 30 s), annealing (56°C, 30 s) and extension (72°C, 40 s), and a final elongation of 10 min at 72°C. Polymerase chain reaction amplicons were assessed by gel electrophoresis in 1% agarose gel run at 100 V for 45 min, and the sizes of the products were validated by comparison with a molecular marker (Mafuna 2019). The PCR products were washed with AMPure XP beads (Beckman Coulter, United States) for library preparation. Library preparation and sequencing were conducted according to Illumina 16S Metagenomic Sequencing Library manual. Thereafter, the prepared libraries were sequenced using the Illumina MiSeq sequencing platform.

#### Data analysis

Raw data from the Illumina MiSeq platform were de-multiplexed, and paired-end fastq reads were pre-processed, filtered and analysed with the DADA2 software package implemented in R and RStudio. The Silva database was used to assign taxonomy to all amplicon sequence variants (ASVs), and taxonomic abundances were visualised using Krona charts.

#### Ethical considerations

All participants gave their informed consent before participating in the study, and ethical clearance was granted by the Durban University of Technology Institutional Research Ethics Committee (ethical clearance no: IREC 027/19). Gatekeepers permission was obtained from KwaZulu-Natal Department of Education.

## Results

### Checklist

#### Demographics

Most respondents were females (who were 25 years old and above) and had at least a secondary education (100%) (Table 1). Of the 140 of the respondents interviewed, approximately 33% received training in the principles and techniques for food hygiene and safety.

**TABLE 1:** Demographic profile of food handlers (*N* = 140).

Variables	Frequency	%
**Gender**		
Female	139	99.3
Male	1	0.7
**Age (years)**		
25–35	22	16.0
36–45	62	44.3
46–55	44	31.0
56–65	12	8.6
**Level of education**		
Completed secondary education (Grade 12)	140	100.0
**Food hygiene training**		
Yes	46	33.0
No	94	67.0

#### Standard requirements for food premises

None of the schools had been issued with a Certificate of Acceptability by EHPs of the local authority. Table 2 shows that 24% of the premises had pest and vector control measures in place. Thirty three per cent provided separate sanitary facilities for food handlers, while hand washing facilities with running water were provided in 39% of premises. Approximately a third of the schools supplied soap for hand washing. Refuse bins with close-fitting lids were provided in approximately 30% of the premises. Hygienic storage facilities for food were available in 55% of premises. An adequate supply of water was available in 88% premises. Pantry food items were stored on shelves or pallets in 58% of the schools, and the shelves or pallets were generally clean and dust-free in 48% of schools. Food contact surfaces were smooth and non-absorbent in 67% of the schools. The surfaces were cleaned before the commencement of each shift in 70% of the schools.

**TABLE 2:** Compliance of schools according to the standard requirements for building structure and storage (*N* = 33).

Standard requirements from R638	No. of compliant schools	%
Premises available and maintained	25	76
All interior surfaces smooth and dust-proof	21	64
Cross-ventilation possible	33	100
Adequate ventilation openings	26	79
Adequate natural light	30	91
Sink with water available	24	73
Pest control in place	8	24
Vector control in place	8	24
Approved waste-water disposal	17	52
Separate toilets for food handlers	11	33
Hand washing facilities available	13	39
Soap and drying mechanism available	12	36
Liquid-proof bin with lid and suitable storage	10	30
Suitable storage space available	18	55
Storage facilities for food handlers	9	27
Adequate water supply	29	88
Fridge was available	2	6
Food not stored directly on the floor	19	58
Food stored on clean shelves	16	48
Working surfaces were made of smooth, rust-proof, non-toxic and non-absorbent material	22	67
Crockery and utensils in good condition	18	55
Work surfaces cleaned and washed before shift	23	70

#### Food handlers

Observations of food handler practices using a standardised checklist are presented in Table 3. Observation of 140 food handlers was carried out using a checklist. Although only 45% of food handlers were provided with suitable protective clothing, 82% were clean at the beginning of their shift. Despite inadequate training, 76% of all food handlers did not wear jewellery while handling food and 61% had short and clean fingernails. Hands were washed regularly by only 56% of food handlers, which was a cause for concern.

**TABLE 3:** Observations of food handler practices (*N* = 140).

Standard requirements	Satisfactory observation	%
Food handlers wore protective clothing	63	45
Food handler adequately trained	46	33
No jewellery while handling food	106	76
Hands and clothes were clean	115	82
Fingernails clean	85	61
Hands washed regularly	78	56
No sores on hands	140	100

### Metagenomic results

**FIGURE 1:**
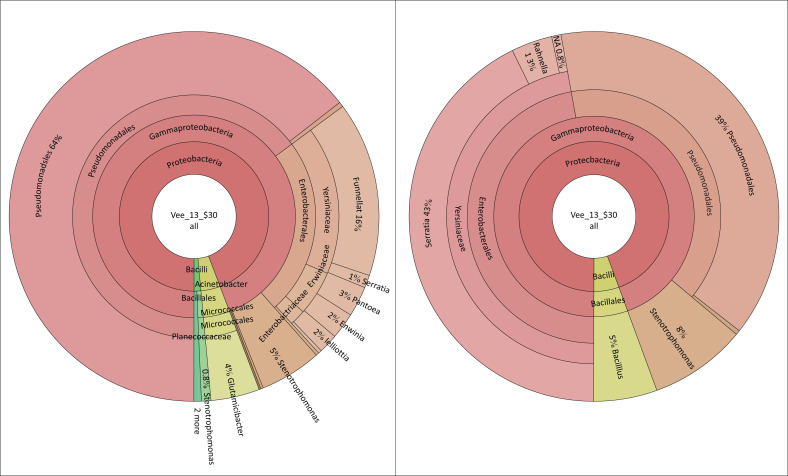
Microbiological results of food contact surfaces and hands of food handlers of School A.

Metagenomic data analysis was performed on swabs to determine the native microbial community associated with NSNP food contact surfaces. Sampling was carried out in the schools that scored the highest (A and D) and lowest (C and D) in the checklist. In school A (Figure 1), *Pseudomonas* sp. (64%), *Serrattia* sp., (43%), *Rahnella1* sp. (16%), *Stenotrophomonas* sp. (8%), *Bacillus* sp. (5%), *Glutamicibacter* sp. (4%), *Pantoea* sp. (3%), *Erwinia* sp. and *Lelliottia* sp. (2%) and *Sporosarcinia* sp. (0.8%) were detected on food contact surfaces. The observation survey revealed that the food preparation area of school A had an overall compliance of 30% and was non-compliant in all areas of the assessment, notably lacking in hand washing facilities, separate sanitary facilities for food handlers and designated food storage. Refuse bins were also not provided, and food was stored in direct contact with the floor. The food contact surfaces were neither made of smooth, non-absorbent material nor cleaned at the beginning of each shift. The food handlers were not provided with protective clothing, 67% had long fingernails and nail polish and none had received any training in food safety and hygiene.

**FIGURE 2:**
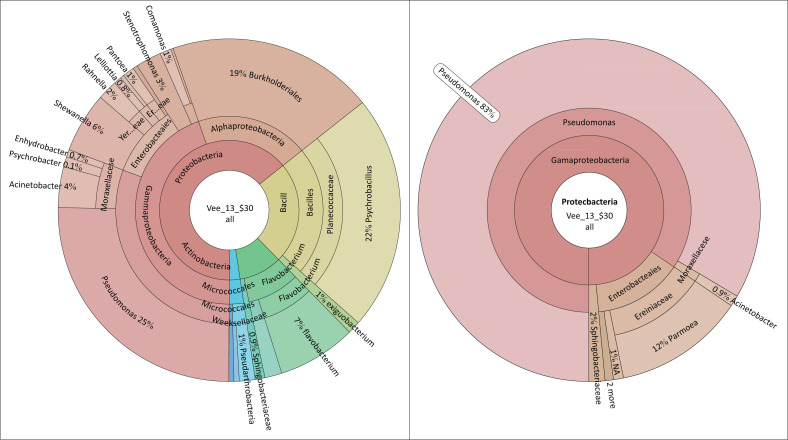
Microbiological results of food contact surfaces and hands of food handlers of School B.

In school B (Figure 2), *Pseudomonas* sp. (83%), *Psychobacillus* sp. (22%), *Brevundimonas* sp. (19%), *Pantoea* sp. (12%), *Flavobacterium* sp. (7%), *Acinetobacter* sp. (4%), *Stenotrophomonas* sp. (3%), *Chryeobacterium* sp. (2%), *Rahnella1* sp. *Comamonas* sp. and *Exiguobacterium* sp. (1%) were detected on the food contact surfaces. The observational survey revealed that the food preparation area of school B had an overall compliance of 70%. This school was partially compliant with the structural, facilities and storage criteria of the assessment, although the school did not have plumbing or separate sanitary facilities for food handlers. A makeshift hand washing mechanism in the form of ‘tippy taps’ was provided to promote hand washing after using the toilet. The food contact surfaces were made of smooth and non-absorbent material, and they were cleaned at the beginning of each shift. The food handlers were provided with protective clothing and had received training in food safety and hygiene.

**FIGURE 3:**
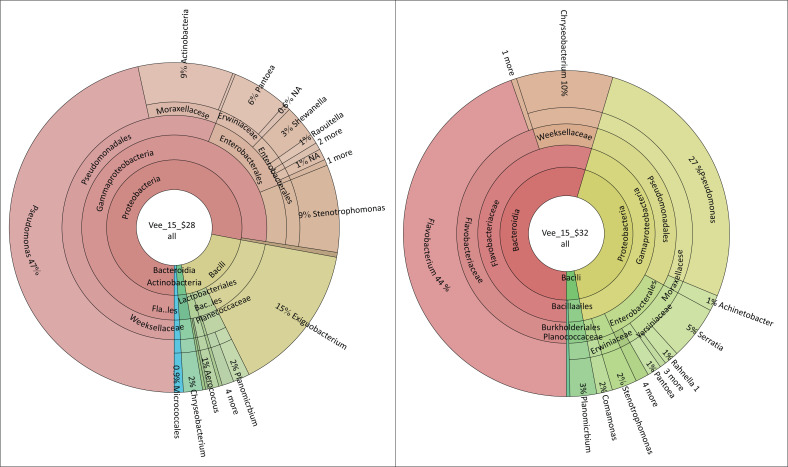
Microbiological results of food contact surfaces and hands of food handlers of School C.

In school C (Figure 3), *Pseudomonas* sp. (47%), *Flavobacterium* sp. (44%), *Exiguobacterium* sp. (15%), *Chryseobacterium* sp. (10%), *Acinetobacter* sp. (9%), *Stenotrophomonas* sp. (9%), *Pantoea* sp. (6%), *Serrattia* sp. (5%), *Shewanella* sp. (3%), *Planomicrobium* sp. (3%), *Chryseobacterium* sp. (2%), *Planomicrobium* sp. (2%), *Aerococcus* sp. (1%) and *Raoutella* sp. (1%) were detected on the food contact surfaces. The observation survey revealed that the food preparation area of school C had an overall compliance of 80%, and it was partially compliant in all areas of the assessment. The school had separate sanitary facilities for food handlers and hand washing facilities in the food preparation area and in the toilet to promote hand washing before the commencement of the shift and after using the toilet, respectively. The food handlers were provided with protective clothing and had received training in food safety and hygiene.

**FIGURE 4:**
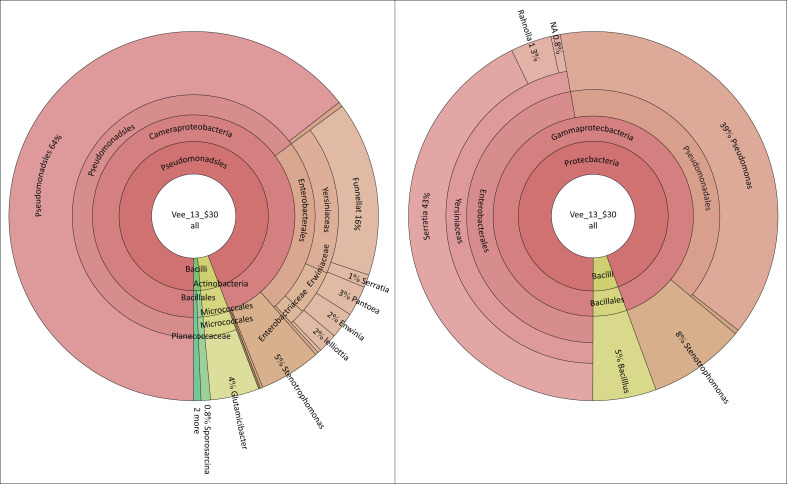
Microbiological results of food contact surfaces and hands of food handlers of School D.

In school D (Figure 4), *Pseudomonas* sp. (84%), *Shewanella* sp. (18%), *Acinetobacter* sp. (16%), *Stenotrophomonas* sp. (15%), *Erwinia* sp. (7%), *Pantoea* sp. (6%), *Raoultella* sp. (4%), *Serrattia* sp. (3%), *Pedobacter* sp. (2%), *Enterobacter* sp. (1%), *Kosakonia* sp. (1%), *Massillia* sp. (1%) and *Stenotrophomonas* sp. (0.9%) were detected on the food contact surfaces. The observation survey revealed that the food preparation area of school D had an overall compliance of 30% as it was non-compliant in all areas of the assessment, especially in the building and storage requirements. The roof of the food preparation area was not dust-proof, ventilation was poor, and hands were washed in a plastic basin where the water was shared by all food handlers. The school did not have a pest control programme in place, and there was no designated food storage. The food contact surfaces were absorbent and were not cleaned at the beginning of the work shift. The food handlers were provided with protective clothing, had been trained in food safety and hygiene, but it was noted that some had long fingernails and there was no soap provided to promote hand washing. The food handlers also shared the sanitary facilities with the educators, and hand washing facilities were not provided.

## Discussion

The aim of this study was to assess the compliance of food preparation and storage areas in primary schools offering NSNP meals to South African Regulation 638 of the *Foodstuffs, Cosmetics and Disinfectants Act* (*No. 54 of 1972*) and to evaluate the resident bacteria present on food handlers and contact surfaces of schools. Compliance to the provisions of the regulation determines the quality of the NSNP meals offered to learners. The findings revealed that the majority of the schools’ food preparation (64%) and storage areas (55%) were only partially compliant with the regulated standard requirements for food premises. The five most common non-compliances in the 33 schools surveyed were poor pest and vector control, inadequate provision of sanitary and hand washing facilities for food handlers, inadequate waste management and the lack of training for food handlers. Observational results suggest that there are several structural shortfalls that need to be addressed to make sure that meals served at schools are cooked and stored in a clean and safe manner. Metagenomic sequencing revealed that *Pseudomonas* sp. (25% – 84%), followed by *Stenotrophomonas* sp. (0.9% – 15%), *Acinetobacter* sp. (0.9% – 16%), *Pantoea* sp. (1% – 12%) and *Serrattia* sp. (1% – 5%) were the common species found on various food contact surfaces in the schools sampled in this study.

Schools have often been implicated as one of the sources contributed to FBD outbreaks with risk factors including (Ababio & Lovatt 2015) improper time-temperature control, improper food handling practices and poor personal hygiene as well as improper storage (Barjaktarović-Labović et al. 2018), inadequate cooking and cross-contamination (Kennedy et al. 2005). Despite the presence of various regulations to ensure safe meals are offered in school canteens, safety measures taken during school meal preparation are still inadequate (Pascual et al. 2016). School cafeterias were responsible for the second highest number of reported FBD outbreak cases (14 163 cases, 23%) in China from 2003 to 2008 (Wu et al. 2018). Data from Ghana have shown that learners who had contracted a foodborne illness during the academic year had a considerably greater rate of sickness than those who had not, and the prevalence of foodborne infections was higher than the annual incidence of 1 in every 40 Ghanaians (Ministry of Food and Agriculture & World Bank 2007). Similarly, Bankolé et al. (2012) reported that unsanitary circumstances at boarding schools contributed to unhygienic meals in Berlin.

Regulation 638 of 2018 (regulations governing general hygiene requirements for food premises), promulgated under the *Foodstuffs, Cosmetics and Disinfectants (FCD) Act 54 of 1972* of the Republic of South Africa, states that food may not be handled on premises without a current Certificate of Acceptability. In South Africa, the Certificate of Acceptability is issued to premises that are fully compliant with all the minimum requirements of R638. In this study, it was revealed that none of the participating schools had been issued with a Certificate of Acceptability. However, these premises continued to operate because the district municipality did not have authority to enforce the regulations of the *FCD Act of 1972*, and the responsible EHPs were not designated as law enforcement officers. This finding was corroborated by a study evaluating the sanitary conditions and food handlers’ practices in the Philippines, where the majority of the canteens of selected public and private schools were not in possession of health cards, such as sanitary permits (Ministry of Food and Agriculture & World Bank 2007; Pascual et al. 2016). The majority of the schools in this study (76%) had designated food preparation areas, which was consistent with the results of Rendall-Mkosi, Wenhold and Sibanda (2013) whose evaluation of the NSNP in Mpumalanga revealed that approximately 85% of schools prepared meals in designated food preparation areas. The lack of appropriate infrastructure and equipment in food service establishments has been the most important issue in food safety (Lockis et al. 2011). Gas was used to prepare food in all the participating schools in Vryheid, and although a fire extinguisher was available, none of the food handlers were trained in fire safety. Fortunately, 79% of the food preparation areas had windows that allowed cross-ventilation, which was essential when cooking with gas (Department of Basic Education 2011).

This study identified poor pest and vector control in many of the schools surveyed. Pests may act as carriers for various bacteria, including foodborne pathogens as they traverse different environments and can deposit onto food contact surfaces (Da Costa et al. 2006), such as chopping boards and dishes (Simothy, Mahomoodally & Neeto 2018). Sealing cracks and crevices and storing food in sealed containers are measures that can be used to control pests and vectors (Sarwar 2015).

Designated sanitary facilities were available in 33% of the schools, and hand washing facilities were provided in only 39% of the surveyed schools. This was consistent with a study assessing the minimum requirements of water, sanitation and hygiene (WASH) in rural schools in Kenya where the World Health Organization (WHO) and United Nations Children’s Fund (UNICEF) (2018) found inadequate provision of water and sanitation. Many diseases are attributable to a lack of water and sanitation; therefore (WHO/Europe 2019), restroom availability and hand washing stations are critical to preventing disease outbreaks (Guzewich & Ross 1999). The data show that many schools in South Africa do not comply with the regulations governing the hygienic standards for food establishments. Until EHPs are authorised to enforce food regulations in this local authority, they act only in an advisory capacity and the relevant department should address the resource and infrastructure issues in the school nutrition programmes and ensure that all applicable food safety regulations are complied with (Department of Health 2018; Singh et al. 2016).

Most food handlers had short, clean nails (61%) and had removed jewellery before commencing with their duties (76%). Sub-regulation 11 of regulation 638 states that a person must wash their hands thoroughly with soap and water right before the start of each work shift, right after a break and before touching equipment. Fingernails must also be short, clean and free of any adornments, and hands should be washed after visiting the latrine and every time they have blown their nose or touched their nose or mouth (Department of Health 2018). A study examining the role of contributing factors and the spread of FBD in school foodborne outbreaks showed that disease outbreaks generally involved food handlers (Venuto, Garcia & Halbrook 2015). Microorganisms on food handlers’ hands enhanced the likelihood of contamination; therefore, practicing good personal hygiene is imperative in ensuring the preparation of food that is safe for consumption by children, who may have an increased highly susceptibility to disease.

Conventional microbiological analysis (selective media, agar and pre-enrichment broth) of food contact surfaces limits the identification of bacteria to those used as indicators of poor hygienic conditions (Da Vitória et al. 2018), thus underestimating bacterial counts (Ríos-Castillo, Ripolles-Avila & Rodríguez-Jerez 2021). The bacteria commonly reported to be implicated in FBDs include *Salmonella spp, S. aureus, E. coli* and *Shigella spp.* (World Health Organization 2014), which are the most prevalent gastrointestinal disease-causing pathogens in sub-Saharan Africa (Fletcher, Stark & Ellis 2011). *Salmonella* has continued to be the most commonly identified causal agent in foodborne illnesses in European nations (22.5% of total outbreaks), and outbreaks associated with *Salmonella* have been traced to worker handling and contaminated food contact surfaces (Abdul-Mutalib et al. 2015). In Mpumalanga, (SA), an evaluation of pathogenic organisms on food contact surfaces in school kitchens showed the highest incidence of *S. aureus* on cutting boards (31.3%) and dry storage shelves (37.5%), followed by benchtops (25%) and refrigerator handles (25%). Although conventional methods for detecting foodborne pathogens have advantages, reliance on culturing microorganisms on agar plates followed by biochemical identification is time-consuming and labour-intensive (Mandal et al. 2011). The process involves multiple steps, including pre-enrichment, selective enrichment and selective plating, which all add to the overall time required for identification. Moreover, the incubation periods needed for bacterial growth on agar plates contribute to the delay in obtaining results. Additionally, the preparation of culture media, inoculation of plates and colony counting further intensify the labour involved in these methods (Zhao et al. 2014). Overall, while conventional methods have been widely used and established, there is a growing need for alternative approaches that can provide faster and more efficient detection of foodborne pathogens.

Microbial communities in food preparation areas are more complex than traditional microbiological assessments suggest, and this study, through metagenomic analysis, highlights the diversity of the microbial community on food contact surfaces and hands of food handlers in the school setting. This study revealed that structural compliance did not translate to the environment being safe for food handling. School C revealed the most diverse microbial community on food contact surfaces. Overall, the most abundant bacterial species in this study was *Pseudomonas*, which was detected on both food handlers’ hands and food contact surfaces. *P. aeruginosa* is one of the most common causative agents of food contamination, and this is of public health concern (Nahar et al. 2021). It is an opportunistic pathogen and a common cause of spoilage in a wide range of vegetables, milk and meat products (Raposo et al. 2016). It has the ability to create biofilms that enable it to cling to processing surfaces for extended periods of time and play a significant part in the cross-contamination of food during handling and processing (Lim et al. 2021). The presence of *Pseudomonas* was an indicator of cross-contamination, poor hand hygiene, faecal contamination and poor water quality (Akusu, Kiin-Kabari & Mwemedo 2016).

The presence of *Stenotrophomonas* (0.9% – 15%), which are commonly isolated from soil, plants, water and raw milk (Wisplinghoff, Seifert & Steven 2014), indicated a failure in the cleaning and disinfection process. Therefore, extensive hygiene practice is recommended in schools. The genus Acinetobacter (0.9% – 16%), typically present on a variety of foodstuffs, particularly refrigerated fresh foods, was expected; however, Ababneh, AL-Rousan and Jaradat (2022) suggest that if it spreads into food preparation areas, it may form biofilms on surfaces, thus becoming a persistent source of contamination in the food chain. It should be noted that 75% of the schools in this study did not have refrigerators. Although rarely considered a pathogen, the *Pantoea* (1% – 12%) has been widely known in both the pre- and post-harvest stages of fruit as a biological control agent (Grimont & Grimont 2005; Nunes et al. 2001). *Pantoea* produces toxin(s) with a broad antimicrobial range, and successful spread was affected by temperature control (Johnson et al. 2000; Völksch & Sammer 2008). The bacterial community in a food processing environment may differ by various environmental factors, and in this study (Lim et al. 2021), the results of the various food contact surfaces highlighted the complexity of the bacterial community, which is normally limited to more common food pathogens. The presence of bacterial pathogens, despite compliance to the standard requirements for food contact surfaces, demonstrated the need for proper hygiene and regular cleaning of food preparation facilities and equipment, and the promotion of personal hygiene to prevent cross-contamination. The research revealed that the bacterial diversity associated with food contact surfaces is broader than the commonly investigated food pathogens. Metagenomics, which has been primarily used in dairies and butcheries, enables bacterial community mapping in food handling or processing facilities, therefore enhancing the identification and subsequent control of the bacterial community on food contact surfaces (Emamjomeh et al. 2023). This work has demonstrated that these environments are inhabited by a resident microbiome that endures despite cleaning procedures and may be easily transferred to the final product (De Filippis et al. 2021).

It is a requirement in the food production environment that continuous improvement opportunities are provided for food handlers. Training should be offered every 6–12 months, and its efficacy should be evaluated. However, only 33% of the food handlers had received previous food safety training provided by EHPs in the previous year. Rendall-Mkosi et al. (2013) also found that some voluntary food handlers (VFHs) in the Eastern Cape were not trained regularly. Additionally, Sibanyoni and Tabit (2019) conducted research revealing that only 27% of the schools in Mpumalanga employed trained food handlers. Sub-regulation 10 of R638 states that it is the duty of a person in charge of food premises to ensure that they, and any other person working on the premises, are adequately trained in the principles of food safety and arrange follow-up training as applicable (Department of Health 2018). As a consequence of the lack of training, food handlers have been found to have poor food safety knowledge and unsafe food handling skills in Malaysia (Sani & Siow 2014; Shinbaum, Crandall & O’bryan 2016). The lack of trained food handlers should be a concern in schools, as NSNP meals are served to children who could easily contract FBDs because of their weak immune systems (Scallan et al. 2013). In Saudi Arabia, 68.1% of foodservice staff in Al Madinah hospitals had received food safety training (Alqurashi, Priyadarshini & Jaiswal 2019). Food establishments in Brazil, such as hospitals (92%) (Ferreira et al. 2014) and schools (93%), have well-trained food handlers (Da Cunha, Stedefeldt & De Rosso 2012), because training courses are legally mandated. Training in hygiene and food safety practices and principles is therefore necessary as it promotes and improves safe handling of food and includes procedures to prevent food contamination and risk of food pathogens. Training needs to be updated and contextual as there may be context limitations related to infrastructure, funding and resources.

## Conclusion

Previous studies regarding microbiological analysis of food contact surfaces targeted specific food pathogens and biomarkers contributing to human diseases, leaving the non-culturable microbial population undetected. Genomic sequencing allows for the identification of intricate microbial communities by analysing their genetic makeup, circumventing issues associated with traditional culturing methods. The data suggest that school food preparation areas were not compliant as none of the participating schools were in possession of a Certificate of Acceptability, which was a legal requirement for all food premises. The majority of the school kitchens surveyed had poor provision of separate sanitary facilities for food handlers, hand washing facilities with running water, refrigeration facilities to prevent spoiling of foodstuffs, shelving to ensure no foodstuffs come in contact with a ground surface, and training of food handlers in the principles of food hygiene and food safety practices. This study is unique as it attempted to detect the variability of bacterial communities on food contact surfaces, especially in the school feeding environment using a metagenomic approach. The hands of food handlers had the most diverse bacterial composition compared to work surfaces. This revealed a need for training of food handlers on food safety principles and personal hygiene, including the importance of providing hand washing facilities with running water and soap. This study reports the various bacterial populations on various types of surfaces in school kitchen surfaces, providing valuable insights for future investigations into the influence of bacterial communities in food processing environments. Investigating the impact of cleanliness and environmental conditions on microbial diversity in food handling settings could offer fresh insights into the relationships between microbes and food systems. This exploration may lead to the development of safer, more efficient and sustainable approaches to food production. Using modern sequencing-based techniques, it is possible to monitor the environmental microbiome of the food sector. This is a potential tool that could assist in comprehensive quality and safety monitoring measures and influence the development of sustainable food hygiene practices.
